# 2013 multistate outbreaks of *Cyclospora cayetanensis* infections associated with fresh produce: focus on the Texas investigations

**DOI:** 10.1017/S0950268815000370

**Published:** 2015-04-13

**Authors:** F. ABANYIE, R. R. HARVEY, J. R. HARRIS, R. E. WIEGAND, L. GAUL, M. DESVIGNES-KENDRICK, K. IRVIN, I. WILLIAMS, R. L. HALL, B. HERWALDT, E. B. GRAY, Y. QVARNSTROM, M. E. WISE, V. CANTU, P. T. CANTEY, S. BOSCH, A. J. DA SILVA, A. FIELDS, H. BISHOP, A. WELLMAN, J. BEAL, N. WILSON, A. E. FIORE, R. TAUXE, S. LANCE, L. SLUTSKER, M. PARISE, the Multistate Cyclosporiasis Outbreak Investigation Team

**Affiliations:** 1 Center for Global Health, Division of Parasitic Diseases and Malaria, Centers for Disease Control and Prevention, Atlanta, GA, USA; 2 Epidemic Intelligence Service, Centers for Disease Control and Prevention, Atlanta, GA, USA; 3 National Center for Emerging and Zoonotic Infectious Diseases, Centers for Disease Control and Prevention, Atlanta, GA, USA; 4 Texas Department of State Health Services, Austin, TX, USA; 5 Fort Bend County Health & Human Services, Rosenberg, TX, USA; 6 United States Food and Drug Administration, College Park, MD, USA

**Keywords:** *Cyclospora*, outbreaks, parasites

## Abstract

The 2013 multistate outbreaks contributed to the largest annual number of reported US cases of cyclosporiasis since 1997. In this paper we focus on investigations in Texas. We defined an outbreak-associated case as laboratory-confirmed cyclosporiasis in a person with illness onset between 1 June and 31 August 2013, with no history of international travel in the previous 14 days. Epidemiological, environmental, and traceback investigations were conducted. Of the 631 cases reported in the multistate outbreaks, Texas reported the greatest number of cases, 270 (43%). More than 70 clusters were identified in Texas, four of which were further investigated. One restaurant-associated cluster of 25 case-patients was selected for a case-control study. Consumption of cilantro was most strongly associated with illness on meal date-matched analysis (matched odds ratio 19·8, 95% confidence interval 4·0–∞). All case-patients in the other three clusters investigated also ate cilantro. Traceback investigations converged on three suppliers in Puebla, Mexico. Cilantro was the vehicle of infection in the four clusters investigated; the temporal association of these clusters with the large overall increase in cyclosporiasis cases in Texas suggests cilantro was the vehicle of infection for many other cases. However, the paucity of epidemiological and traceback information does not allow for a conclusive determination; moreover, molecular epidemiological tools for cyclosporiasis that could provide more definitive linkage between case clusters are needed.

## INTRODUCTION

*Cyclospora cayetanensis* is the protozoan parasite that causes cyclosporiasis. Cyclosporiasis is characterized by profuse watery diarrhoea; anorexia, fatigue, weight loss, nausea, flatulence, abdominal cramping, myalgia, vomiting, and low-grade fever may also occur [1]. Symptoms typically begin an average of 7 days (range 2–14 days) after ingestion of the infective form of the parasite and, if infection is left untreated, may last weeks to months with remitting and relapsing symptoms [1]. Cyclosporiasis occurs most commonly in tropical and subtropical regions. In the United States, at least one third of non-outbreak-associated cyclosporiasis cases have been associated with international travel [2]. Since the mid-1990s cyclosporiasis outbreaks have been linked to imported fresh produce, including raspberries, basil, snow peas, and mesclun lettuce [1, 3, 4]. *C. cayetanensis* is not directly transmitted from one person to another; an infected person sheds unsporulated oocysts in the faeces, which are non-infective. These oocysts do not multiply outside the human host and require days to weeks in the environment to become infective [5]. Infection is seasonal, and the time of year and environmental conditions responsible for transmission vary around the world. In the United States, most of the reported cases have occurred during May–August, peaking in June and July [1, 2].

The 2013 multistate outbreaks of cyclosporiasis contributed to the highest annual number of cyclosporiasis cases in the United States since 1997. Cyclosporiasis has been a nationally notifiable disease in the United States since 1999 [2]. At the time of writing this paper, cases of cyclosporiasis are reportable in 40 states, the District of Columbia, and New York City, which notify the Centers for Disease Control and Prevention (CDC) of cases via the Nationally Notifiable Diseases Surveillance System [6]. On 28 June 2013, CDC was notified of two laboratory-confirmed cases of cyclosporiasis in Iowa residents who became ill in June and did not have a history of international travel during the 14 days before illness onset. After additional cases were reported from other states, collaborative epidemiological and traceback investigations were conducted by CDC, state and local public health officials, and the U.S. Food and Drug Administration (FDA). Three states reported the majority of cases: Texas, Iowa, and Nebraska. The implicated food vehicle in the restaurant-associated cases in Iowa and Nebraska was a prepackaged salad mix, produced by a supplier in Guanajuato, Mexico, containing iceberg and romaine lettuce, red cabbage, and carrots [7]. We describe the investigations in Texas, the state with the highest number of reported cases during the investigation period (1 June–31 August 2013) and where a different food vehicle was responsible for illnesses.

## METHODS

### Ethical approval

The Center for Global Health within the CDC determined that these investigations were in response to a public health threat and did not meet the definition of human subject research, and were therefore exempt from institutional review board approval. In the case-control study conducted, the purpose of the investigation and voluntary nature of participation were explained to all participants and verbal assent was obtained prior to all interviews.

### Definitions

We defined a confirmed case as laboratory-confirmed cyclosporiasis in a person who had onset of illness between 1 June and 31 August, and who did not have a history of international travel during the 14 days before symptom onset. We defined a cluster as more than one unrelated ill person (i.e. people who did not know or live with each other), at least one of whom had a laboratory-confirmed infection, who reported eating at the same restaurant, shopping at the same grocery store, or attending the same event (e.g. social gathering) as a confirmed case-patient in the 2–14 days before becoming ill (the incubation period for *Cyclospora*). Within a cluster, we defined a probable case as diarrhoea (i.e. ⩾3 loose stools in 24 h) in a person who ate food from the same restaurant, grocery store, or event as a confirmed case-patient during the 2- to 14-day incubation period. We further classified a probable case as epidemiologically linked if diarrhoea occurred in a person who ate food with (i.e. dined together in the same group) a confirmed case-patient from the same restaurant within the incubation period.

### Outbreak identification and case ascertainment

The investigators used a hypothesis-generating questionnaire to identify potential vehicles of infection and clusters of cases for further investigation. The Texas Department of State Health Services targeted clusters with ⩾3 case-patients and available epidemiological (e.g. onset and exposure dates) and traceback information (e.g. receipts and shopper card information). One cluster was identified at a Mexican-style restaurant in southeastern Texas (restaurant A). A menu-specific questionnaire was used to identify common food items consumed by case-patients who ate at restaurant A. The questionnaire addressed meal date and time and details about menu items consumed by each patron, including condiments. Case-patients were asked to provide names and telephone numbers of their meal companions, which facilitated identification of epidemiologically linked cases and controls.

Two clusters associated with Mexican-style restaurants (restaurants B and C) in central Texas were also identified; neither restaurant was affiliated with restaurant A or each other. An additional cluster of case-patients from a grocery store in northeastern Texas was also identified. Data from shopper cards were used to identify common produce items purchased.

### Restaurant A case-control study and environmental investigation

To identify the most likely vehicle(s) of infection for the restaurant A cluster, we conducted a case-control study. We defined a control as a person who ate food from the restaurant and reported no diarrhoea during the 14 days thereafter. We identified controls through credit card receipts, which included patron names and meal dates, and through interviews of case-patients, who provided names and contact information for meal companions. We attempted to identify at least three meal date-matched controls per case-patient. Trained personnel conducted telephone interviews using the menu-specific questionnaire.

The environmental investigation included reviewing with management the daily operation of the restaurant, ordering practices and suppliers for the exposure period, and inspecting and evaluating food storage and preparation areas and practices. We interviewed food handlers to assess what meals they ate at the restaurant and if they had been ill. The chef provided an ingredient list for each menu item and recipes for condiments including salsas.

### Traceback investigations

Traceback investigations were conducted to determine common suppliers of produce to the pertinent points of service indicated by the epidemiological investigations. State and local health departments in Texas and CDC contacted the points of service to collect supplier and distributor information, which was forwarded to FDA. FDA district offices contacted the distributors and collected invoices and other records. FDA reviewed records pertaining to purchases made at each level of the distribution chains.

### Statistical analysis

For the restaurant A cluster, data were analysed using SAS v. 9.3 (SAS Institute Inc., USA). To identify associations between cyclosporiasis and meal and condiment ingredients, we conducted unmatched and matched analyses of data from case-patients and controls. For many exposures, the number of persons was too small to use asymptotic methods; therefore, we used an exact method (i.e. exact conditional logistic regression) [8]. Matched case-control sets were treated as strata in these analyses, allowing us to control for the heterogeneity of risk across matched sets. We report the results as odds ratios (ORs) with corresponding 95% confidence intervals (CIs); we provide a median unbiased estimate of the OR if the upper bound of the CI is infinity.

### Laboratory analysis of stool specimens

Stool specimens positive for *Cyclospora* on examination at the Texas State Public Health Laboratory were sent to CDC's Parasitic Diseases Reference Diagnostic Laboratory for confirmatory testing and identification of the parasite to the species level (i.e. *C. cayetanensis*). CDC examined wet mounts of the specimens by ultraviolet (UV) fluorescence microscopy to identify *Cyclospora* oocysts, which are autofluorescent. Because these specimens had been stored in zinc polyvinyl alcohol, they were suitable for molecular analysis. CDC conducted DNA extraction as described previously [9] and adapted a TaqMan real-time PCR method for species-level identification [10]. To ensure the validity of the PCR results, CDC examined a subset of the specimens by DNA sequencing of the 18S rRNA gene as described previously [11].

## RESULTS

### Case reporting for multistate outbreaks

**Fig. 1.**
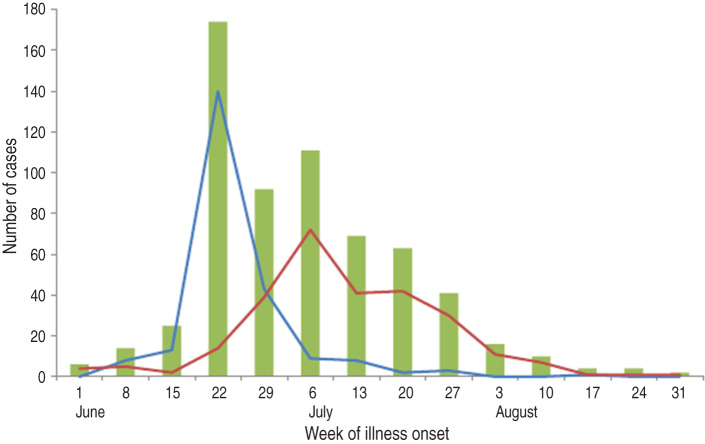
Confirmed cyclosporiasis cases (*n* = 631) reported during the investigation period (1 June to 31 August 2013) by week of illness onset. Total cases in the United States are shown in the green bar graph, Texas cases by the red line, and a combination of Iowa and Nebraska cases by the blue line.

CDC was notified of 631 laboratory-confirmed cases of cyclosporiasis in residents of 25 states whose illness onset dates were during June–August 2013 (Fig. 1). Case-patients had a median age of 52 years (range <1–94 years); 298 (58% of 518) were female. Of the 596 persons with available information, 49 (8%) were hospitalized and no deaths were reported. The three states with the highest numbers of reported cases were Texas (270 cases, 43%), Iowa (140, 22%), and Nebraska (87, 14%). In Iowa and Nebraska, the number of cases peaked in mid-June, whereas in Texas the peak occurred in early July (Fig. 1).

More than 70 clusters of cases linked to multiple restaurants and grocery stores in various regions of Texas were identified. Most (86%) of the clusters involved two or three ill persons. Of the 26 clusters (12 restaurants, 14 grocery stores) with ⩾3 ill persons, only four clusters had adequate epidemiological and traceback information to conduct a thorough investigation. The four clusters investigated are summarized in Table 1 and are described below.

**Table 1.** Summary of investigations of cluster-associated cases of cyclosporiasis in Texas

Cluster (region)	No. of cases	Type of investigations conducted	Exposure dates	Illness onset dates
Restaurant A (Southeastern)	25	Case-control study and fresh produce traceback	6–16 July	10–24 July
Restaurant B (Central)	3	Fresh produce traceback	23 June and 23 July	29 June–26 July
Restaurant C (Central)	6	Fresh produce traceback	26 June–5 July	2–12 July
Grocery store X (Northeastern)	4	Cilantro traceback	22–25 June	27 June–3 July

### Restaurant A case-control study

A total of 25 case-patients were enrolled in the case-control study. Of these, 18 were confirmed cases and seven were probable cases, three of which were epidemiologically linked. The four probable cases that were not epidemiologically linked were identified through credit card receipts.

Meal dates were identified for 21 (84%) case-patients and included five dates (Table 2); the telephone interviews were conducted 4–6 weeks after the reported meal dates. The dates of illness onset were known for 22 (88%) case-patients and ranged from 10 July to 24 July (Table 2). The median incubation period was 7 days (*n* = 18) for both the confirmed (range 4–16 days) and probable (range 6–8 days) cases. We matched by meal date the 21 case-patients whose meal date was known with 65 controls (Table 2).

**Table 2.** Restaurant A case-control study of cyclosporiasis – number of cases and controls by meal date*

		Controls		
Meal date	Cases	Meal companion	Credit card	Total
Saturday, 6 July	8	5	16	21
Sunday, 7 July	1	3	4	7
Friday, 12 July	5	0	16	16
Saturday, 13 July	6	5	13	18
Tuesday, 16 July	1	2	1	3
Unknown	4			
Totals	25	15	50	65

* Restaurant A was closed on Mondays and was closed from 1–4 July for the Independence Day Holiday.

Of 58 ingredients used in preparing food items on the restaurant's menu, ten were significantly associated with cyclosporiasis (Table 3). The only ingredient eaten by all 25 case-patients was cilantro, the ingredient most strongly associated with illness (OR 19·9, 95% CI 4·1–∞). Consumption of lettuce was not associated with illness (iceberg lettuce: matched OR 2·5, 95% CI 0·79–8·4; romaine lettuce: matched OR 3·0, 95% CI 0·16–∞). Neither red cabbage nor carrots (the other components of the salad mix linked to cases of cyclosporiasis in Iowa and Nebraska) were used at restaurant A.

**Table 3.** Frequencies and odds ratios for selected food exposures in case-control study of cyclosporiasis linked to restaurant A

	Unmatched analysis (cases = 25, controls = 65)			Matched analysis (cases = 21, controls = 65)		
Exposure	Cases, *n* (%)	Controls, *n* (%)	OR (95% CI)	Cases, *n* (%)	Controls, *n* (%)	OR (95% CI)
Ingredient-level analysis						
Cilantro	25 (100)	41 (63·1)	19·9 (4·1–∞)	21 (100)	41 (63·1)	19·8 (4·0–∞)
Corn tortilla	24 (96·0)	39 (60·0)	15·7 (2·3–682·8)	20 (95·2)	39 (60·0)	14·4 (2·0–636·0)
Garlic	24 (96·0)	44 (67·7)	11·2 (1·6–493·1)	20 (95·2)	40 (61·5)	15·3 (2·1–697·7)
Salt	24 (96·0)	48 (73·8)	8·4 (1·7–369·8)	20 (95·2)	44 (67·7)	10·7 (1·5–475·4)
Onion	23 (92·0)	40 (61·5)	7·1 (1·5–67·0)	20 (95·2)	48 (73·8)	7·7 (1·1–345·9)
Tomato	23 (92·0)	43 (66·2)	5·8 (1·2–55·2)	19 (90·5)	43 (66·2)	5·5 (1·1–54·1)
Thai arbol pepper	22 (88·0)	30 (46·2)	8·4 (2·2–48·0)	18 (85·7)	30 (46·2)	9·3 (2·2–57·4)
Chili powder*	8 (32·0)	9 (13·8)	2·9 (0·83–10·0)	8 (38·1)	8 (12·3)	5·0 (1·3–22·3)
Corn shucks*	8 (32·0)	8 (12·3)	3·3 (0·93–11·9)	8 (38·1)	9 (13·8)	4·5 (1·1–19·7)
Pork belly	6 (24·0)	2 (3·1)	9·6 (1·6–105·2)	5 (23·8)	2 (3·1)	10·3 (1·4–131·5)
Condiment-level analysis						
Side salsa	19 (76)	27 (41·5)	4·4 (1·4–15·3)	16 (76·2)	27 (41·5)	5·7 (1·6–23·7)
Fire-roasted salsa	18 (72)	29 (44·6)	3·2 (1·1–10·2)	15 (71·4)	29 (44·6)	3·5 (1·1–12·7)
Hot sauce	15 (60)	9 (13·8)	9·0 (2·8–31·1)	12 (57·1)	9 (13·8)	8·0 (2·3–31·4)
Salsa ranchera	5 (20)	5 (7·7)	3·0 (0·61–14·3)	4 (19)	5 (7·7)	6·0 (0·75–75·2)

OR, Odds ratio; CI, confidence interval.

* Not statistically significant in unmatched analysis, but was significant in matched analysis

Restaurant A prepared and served three salsas: side salsa, fire-roasted salsa, and salsa ranchera, as well as one in-house-prepared hot sauce, all of which contained both cilantro and tomatoes. Of these, the side salsa, the fire-roasted salsa, and the hot sauce contained fresh cilantro and were associated with illness (Table 3). The salsa ranchera, which contained only cooked ingredients, was not associated with illness (Table 3). Of note, the side salsa and hot sauce contained canned tomatoes, whereas the fire-roasted salsa contained fresh tomatoes.

The environmental investigation at restaurant A did not identify practices that could result in contamination of the ingredients with *Cyclospora*. All of the interviewed restaurant staff reported eating at the restaurant, but none reported diarrhoeal illnesses during June and July. No food items from the exposure period were available for laboratory testing.

### Two other restaurant clusters

Formal epidemiological studies were not conducted at restaurants B and C because of the small number of cases, three and six, respectively. However, information gathered on the hypothesis-generating questionnaire revealed that all nine case-patients had eaten cilantro in the 14 days prior to symptom onset. Invoices from the two establishments allowed for traceback investigations to be conducted.

### Grocery store cluster

Four additional case-patients independently reported having eaten fresh cilantro they purchased at the same local grocery store before they became ill. Shopper card information was available for three of these persons, all three of whom purchased fresh cilantro within a 4-day period (22–25 June) and developed diarrhoeal illness 5–10 days after their purchase. Cilantro was the only common produce item that all three persons purchased at this grocery store.

### Traceback investigations and control measures

Available traceback information indicated that restaurant A received cilantro from a produce supplier located in Puebla, Mexico during the period of interest. Restaurants B and C and the grocery store were supplied by the same distribution centre. This distribution centre received cilantro from two suppliers located in Puebla, Mexico, different than the supplier to restaurant A. Thus, Puebla was a shared source of fresh cilantro served at all three restaurants and available at the grocery store during the period when case-patients purchased cilantro. Whether the cilantro came from the same location in Puebla or was processed at the same facility could not be determined. As a result of this investigation, FDA increased its surveillance efforts on cilantro products exported to the United States from Mexico [7].

### Laboratory analysis of stool specimens

CDC confirmed the presence of *Cyclospora* oocysts and *C. cayetanensis* DNA by UV fluorescence microscopy and real-time PCR analysis, respectively, in all 66 specimens received from case-patients in Texas. DNA sequencing, which was performed for ten of these specimens, confirmed the PCR results.

## DISCUSSION

The summer 2013 outbreaks represent one of the largest numbers of reported cyclosporiasis cases in the United States since it was added to the list of nationally notifiable diseases in 1999; and contributed to the highest annual number of cyclosporiasis cases since 1997. The highest number of reported outbreak-associated cases of cyclosporiasis since it became nationally notifiable was 692, which occurred in 2005 [12]. Since then, between 2006 and 2011, the median annual number of reported cases of cyclosporiasis has been 140 (range 93–179) [13], which typically includes travel-associated and outbreak cases. Between 2000 and 2011 there was a median of 21 cases (range 9–151) of cyclosporiasis reported in the United States from June to August.

Cilantro was the most likely vehicle of infection in restaurant A, B, C, and grocery store clusters. The strong positive association between cilantro consumption and illness and the data from ingredient-level analyses of the salsa in the case-control study, along with the consistency of cilantro exposure among the case-patients in all four investigated clusters, provide compelling evidence of cilantro as the vehicle of infection. Although a thorough investigation could not be conducted on the remaining clusters in Texas, the temporal association of these four clusters with the large overall increase in cyclosporiasis cases in Texas suggests cilantro was the vehicle of infection for many other cases in Texas.

In Table 3, the ORs for corn tortilla, garlic, and salt are high; this is due to the effect of confounding. These three items are confounded with cilantro probably because they all measure the same latent variable – whether or not a person ate chips and side salsa. All patrons of restaurant A were served chips, made with corn tortillas, and side salsa upon arrival at the table. Notably, garlic and salt were ingredients used in the preparation of most of the menu items served in restaurant A. Moreover, these three items are not biologically plausible vehicles of infection for *Cyclospora*. Historically, foodborne outbreaks of cyclosporiasis have been associated with fresh produce that does not have an outer shell that needs to be removed prior to consumption.

Restaurant-associated illnesses in Iowa and Nebraska during June 2013 were linked to a prepackaged salad mix from a supplier in Guanajuato [14], whereas the fresh cilantro linked to the Texas clusters was grown in Puebla [7]. Therefore, there were at least two US outbreaks of cyclosporiasis associated with different food vehicles from different regions of Mexico during summer 2013.

The occurrence of multiple outbreaks of foodborne cyclosporiasis linked to different types of fresh produce during a single season is not unprecedented. During March to July 1997, three separate and unrelated US outbreaks of cyclosporiasis occurred, which were linked to mesclun lettuce from Peru, raspberries from Guatemala, and basil from multiple possible sources [1].

During 2011 and 2012, CDC assisted with two additional investigations in which cilantro was a likely vehicle of infection for *C. cayetanensis*. In 2011, the Florida Department of Health investigated a restaurant-associated outbreak of 12 cases of cyclosporiasis [15]. Epidemiological and environmental investigations suggested cilantro and onions as the most likely vehicles of infection, because these two ingredients were used in all meals eaten by case-patients. Unfortunately, traceback investigations could not be performed because invoices and receipts were not available [15]. In 2012, state and local health departments in Texas investigated cases of cyclosporiasis associated with a Mexican-style restaurant. A case-control study of 16 cases and 12 controls suggested that fish tacos, containing cabbage and cilantro, were linked to infection. However, a vehicle was not conclusively identified and traceback investigations could not be completed. Most recently, in 2014, investigations conducted of Mexican-style restaurant clusters in Texas linked illnesses again to cilantro imported from Puebla, Mexico [16, 17].

Investigations of foodborne outbreaks in general and cyclosporiasis specifically pose many challenges. In general, patients may not recall the pertinent food exposure that occurred weeks to months before they are interviewed, and typically the food items are no longer available for testing once an investigation is under way [1, 18]. Additionally, many fresh produce items are served as garnishes, incorporated into a mixture with other plausible ingredients (e.g. salad mix and fruit salad), or used in other ways that are inconspicuous. Specific to cyclosporiasis is the relatively long incubation period compounded by delays in diagnosis and reporting of cases that may impede the recognition of an outbreak. Moreover, in most laboratories, routine stool analysis for ova and parasites may not include the examination for the presence of *Cyclospora* oocysts; such testing must be specifically requested, leading to delays in diagnosis. In our experience UV fluorescence microscopy of unstained wet mounts is sensitive and significantly less time-consuming and labour-intensive. However, molecular methods are required to characterize the parasite to the species level.

Determining whether cases of cyclosporiasis are linked to each other and to particular food items is a major challenge. For example, we could not determine the vehicle of infection for the majority of cases in the Texas investigation; the paucity of data to link these cases precluded formal epidemiological investigations. The outbreaks of cyclosporiasis in 2013 underscore the need for molecular subtyping to complement evidence from epidemiological investigations, potentially assisting in identifying the number of outbreaks in a given season and suggesting links between clusters, and facilitating source tracking. Molecular methods that can differentiate *C. cayetanensis* to the haplotype or genotype level have yet to be developed [19]. This research is limited by the difficulty in obtaining *C. cayetanensis* oocysts/DNA for analysis, further compounded by the need to obtain and analyse specimens from geographically diverse settings. Of note, infected humans are the only known source of the parasite, and no methods to reproduce these oocysts *in vitro* or *in vivo* have been established [1]. If candidate subtyping markers are identified in the future, retrospective analysis of stool specimens, such as those that CDC tested by molecular methods in 2013, could be useful in the validation process of potential genotyping methods.

An increasing number of foodborne outbreaks have been associated with the consumption of fresh produce [20–22]. Reducing foodborne illness linked to produce consumption continues to be a difficult task for public health and the produce industry. The specific challenges posed by *Cyclospora* include under-detection of cases, lack of subtyping methods to link cases to each other or to specific food items, and the absence of practical tools to detect the organism in food and potential sources of contamination in the environment (e.g. soil and insanitary irrigation water). Advances in these areas may assist in better understanding the biology of *C. cayetanensis*, the epidemiology of cyclosporiasis, and aid in establishing control measures to reduce contamination of fresh produce.

## APPENDIX

### Cyclosporiasis Outbreak Investigation Team

C. B. Behravesh, T. Benedict, C. Braden, M. de Almeida, J. Jones, A. Kerk, C. Kiebler, B. Mathison, S. Montgomery, C. Schwensohn (*Centers for Disease Control and Prevention, Atlanta, GA*); T. Baker, C. Davis, N. Drake, G. Gana, C. Lozano, K. Klein, D. Olinger, K. Reynolds, A. Ryder (*Fort Bend County Health & Human Services, Rosenberg, TX*); T. Lee (*City of Rosenberg Health Department, Rosenberg, TX*).
